# 

**Published:** 1898-05

**Authors:** 


					﻿Vol. XVIII.
MAY, 1898.
No. 5?*
THE
pOMlEOPATHlC pHY^lClAfl,
A Monthly Journal of Homoeopathic Materia Medica
and Clinical Medicine.
“ He is a freeman whom truth makes free, and all are slaves beside."
"Seek the truth: come whence it may, cost what it will."
- EDITED AND PUBLISHED BY
WALTER M. JAMES, M. E>.
PHILADELPHIA :
TSTo. 1231 LOCUST STREET.'
- Yearly Subscription, $2.50, invariably in advance, Single nuenbcrs9 25 etc.
Entered at the Philadelphia Poet-Office as Second-Claai Mail Matter.
				

